# Event-related brain potential and postural muscle activity during standing on an oscillating table while the knee, hip, and trunk are fixed

**DOI:** 10.1186/s40101-016-0088-4

**Published:** 2016-02-18

**Authors:** Katsuo Fujiwara, Mariko Irei, Naoe Kiyota, Chie Yaguchi, Kaoru Maeda

**Affiliations:** 1 Department of Sports and Health, Kanazawa Gakuin University, 10 Sue-machi, Kanazawa, 920-1392 Japan; 2 Department of Rehabilitation Science, Osaka Health Science University, 1-9-27 Tenma, Kita-ku, Osaka 530-0043 Japan; 3 Department of Rehabilitation, Japan Health Care College, 6-17-3 Megumino-nishi, Eniwa, 061-1373 Japan; 4 Department of Physical Therapy, Faculty of Human Science, Hokkaido Bunkyo University, 5-196-1, Kogane-chuo, Eniwa, 061-1449 Japan; 5 Department of Physical Therapy, Morinomiya University of Medical Sciences, 1-26-16 Nanko-kita, Suminoe-ku, Osaka 559-8611 Japan

**Keywords:** Floor oscillation, Event-related brain potential, Postural control, Joint fixation

## Abstract

**Background:**

In this study, a cast brace was used to immobilize the knee, hip, and trunk, and relations between the event-related brain potential (ERP) and postural muscle activity were investigated while standing on an oscillating table.

**Methods:**

Twelve healthy young adults maintained a standing posture for 1 min per trial while oscillating in the anteroposterior direction at 0.5 Hz with a 2.5-cm amplitude. Trials were performed without and with the cast brace (no-fixation and fixation condition, respectively) until the subject had adapted to the floor oscillation. The ERP from the Cz electrode, postural muscle activity, and joint movement range were analyzed for the first and last two trials (before and after adaptation, respectively).

**Results:**

Movement range of the hip and knee was lower in the fixation condition than in the no-fixation condition, and postural control was achieved by pivoting at the ankle. Peak muscle activity was largest in the gastrocnemius (GcM) in both conditions. GcM activity significantly increased after fixation and then decreased with adaptation. The time of peak erector spinae (ES) activity in the fixation condition was significantly earlier than in the no-fixation condition and was not significantly different from the time of the anterior reversal and peak of triceps surae activity. The negative ERP peaked approximately 80 ms after the anterior reversal. Significant correlations between the time of the peak negative ERP and the peak GcM, soleus, and ES activity were observed only after the adaptation, and were greater in the fixation condition (*r* = 0.83, 0.84, and 0.83, respectively) than in the no-fixation condition (*r* = 0.62, 0.73, and 0.51, respectively).

**Conclusion:**

All joints of the leg and trunk except for the ankle were rigidly fixed by the cast brace, and the phase differences between body segments were very small in the fixation condition. High correlations between the time of the peak negative ERP and the peak GcM, soleus, and ES activity after adaptation in the fixation condition suggest that attention would be more focused on anticipatory processing of muscle sensory information from the triceps surae and/or ES, particularly GcM, which had the greatest activation.

## Background

Many researchers have investigated the postural responses to external perturbations while standing to resolve the dynamic characteristics of the postural control system [1–4]. When a periodic sinusoidal floor oscillation is used as a postural perturbation, subjects continually acquire sensory feedback related to the balance disturbance and easily anticipate the disturbance timing, direction, and magnitude. Floor oscillations can be used to investigate postural control adaptation because postural stability rapidly improves [5–8]. In the floor oscillation task with eyes closed, with higher frequency oscillation, a clearer slow-drift of head was observed, which suggests that vision contributes to the maintenance of head position in space [4, 9]. In the repetition of floor oscillation at a 0.5-Hz frequency with eyes closed, the sway of higher body parts was adaptively decreased compared with other body parts (1/2 before adaptation), and cross-correlation coefficients between sway of higher body landmarks and floor movement became much lower [10]. In addition, we have previously observed that variability of the electrooculogram (EOG) waveform was extremely small [11], suggesting that there was little head rotation [12]. These suggest that vestibular information would not strongly contribute to the perception of floor position during oscillation. In many healthy young adults, posterior muscles, particularly the triceps surae (TS), are dominantly activated as adaptation progresses. The maximum activity of posterior muscles occurs around the anterior reversal of the oscillation, and the minimum activity occurs around the posterior reversal [8, 11, 13]. Therefore, after adaptation, many subjects should prepare for postural control with a focus on the anterior reversal and direct their attention to sensory information from the posterior muscles, particularly the TS, near the anterior reversal.

To examine this hypothesis, we have previously measured event-related brain potentials (ERPs), including the contingent negative variation and readiness potential, during floor oscillations with eyes closed [11]. With sufficient adaptation to the floor oscillation, the negative potential of the ERP gradually increased in magnitude from the posterior reversal to the anterior reversal and had a relatively sharp negative peak at about 80 ms after the anterior reversal. In a previous study of transient postural perturbations [14–16], the ERP had a negative peak approximately 100 ms after the perturbation, and this was considered to reflect the processing of sensory information related to the postural disturbance. When subjects directed attention toward the posterior reversal of the floor oscillation, by performing finger flexion in accordance with that point, the negative ERP peak was observed around the posterior reversal [11]. In addition, a significant correlation (*r* = 0.46) was observed between the latencies of the ERP peak and the gastrocnemius (GcM) electromyogram (EMG) peak [11]. Therefore, the ERP peak during floor oscillations is assumed to reflect the processing of sensory information from the GcM generated near the anterior reversal and the aspects of directed attention.

However, it has been reported that there are inter-individual differences in body sway and postural muscle activity during floor oscillations. For body sway, the phase delay of the higher body parts relative to the floor oscillation varies across individuals [9, 11]. In addition, for some subjects, thigh and/or trunk muscles showed comparatively large activation [10]. These individual differences are likely due to the many degrees of freedom in joints and muscles available to maintain upright standing [17]. Some subjects might strongly direct their attention to sensory information from muscles other than the TS or from joint receptors other than the ankle. Thus, to focus the postural control target on the muscles around the ankle, we fixed all joints of the leg and trunk above the ankle [18]. After sufficient adaptation of postural control pivoting at the ankle in this condition, the attention would be focused on the sensory information related to the ankle movement, especially the muscular sensory information from the TS [19].

In the present study, we fixed the knees, hips, and trunk using a cast brace and investigated the relation between postural muscle activity and ERP during standing on an oscillating table. The working hypotheses were as follows: after sufficient adaptation to the postural disturbance with joint fixation, (1) postural control would be achieved by pivoting at the ankle and (2) there would be a very strong correlation between the latency of the peak ERP and peak EMG activity of the TS, which would both occur near the anterior reversal point of the floor oscillation.

## Methods

### Subjects

Subjects were 12 healthy young adults (five men, seven women). Mean (standard deviation) age, height, weight, and foot length (FL) were 20.4 (1.7) years, 164.6 (7.0) cm, 59.7 (5.2) kg, and 24.2 (1.5) cm, respectively. No subject had any history of neurological or orthopedic impairment. In accordance with the Declaration of Helsinki, all subjects provided informed consent after receiving an explanation of the experimental protocol, which was approved by an ethics committee of Kanazawa University (No. 946).

### Apparatus and data recording

**Fig. 1:**
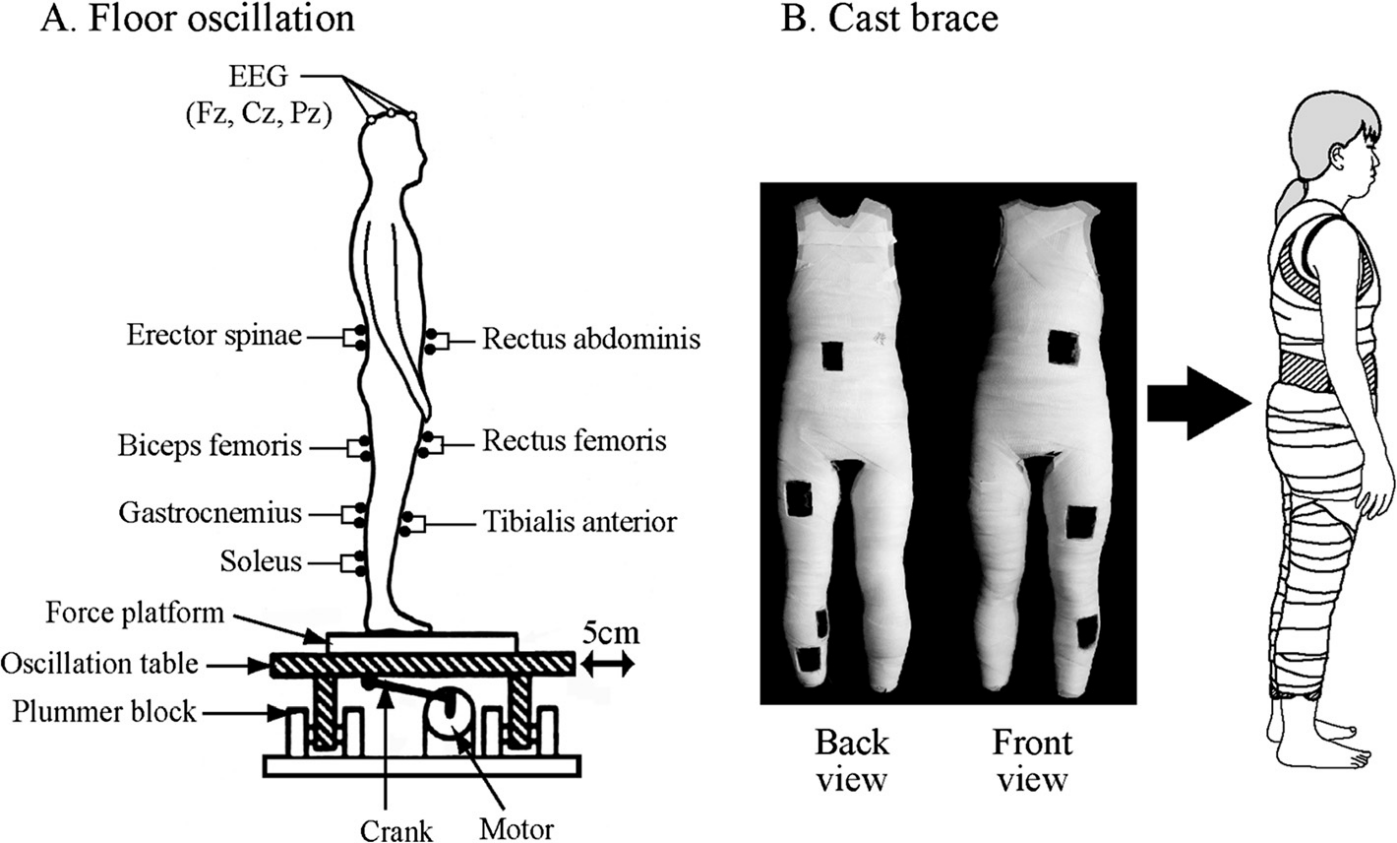
Experimental setup. **a** Floor oscillation. **b** Cast brace. There were seven openings in the cast brace for the EMG electrode wires. The cast brace was attached while standing

A force platform (50 cm long and 50 cm wide, FPA34, Electro Design, Japan) consisting of four load cells was used to record the center of foot pressure in the anteroposterior direction (CoPap). An oscillation table (PW0198, Electric Control Group, Japan) attached to the force platform was oscillated sinusoidally at 0.5 Hz and with a 2.5-cm amplitude in the anteroposterior direction (Fig. 1a). The action point (pressure center) to the floor was consistent with the theoretical calculation when a rigid body with the center of gravity at a certain height was oscillated (error, 5 %) [2]. Thus, the validity of this CoPap fluctuation measurement by the force platform was verified. The table oscillation curve and frequency were measured using a linear position sensor (LP10, Midori Electric, Japan) and a frequency counter (TR-5822, Advantest, Japan), respectively. Earplugs were worn to minimize auditory noise from the oscillating table.

Ag-AgCl cup electrodes (8 mm in diameter) for electroencephalogram (EEG) recording were affixed to the scalp at Fz, Cz, and Pz in accordance with the International 10–20 system and referred to linked ear lobes. A ground electrode was placed at Fpz. An EOG was recorded from a pair of electrodes placed above and below the left eye to monitor artifacts accompanying eye movement. Ag-AgCl surface electrodes (30 mm in diameter, P-00-S, Ambu, Denmark) were used in a bipolar derivation to record surface EMG from the following muscles on the left side of the body: rectus abdominis (RA) at the level of the navel, erector spinae (ES) at the level of the iliac crest, rectus femoris (RF) at the midpoint between the anterior inferior iliac spine and upper border of the patella, long head of biceps femoris (BF) at the midpoint between the ischial tuberosity and head of the fibula, tibialis anterior (TA), medial head of GcM, and soleus (Sol). Electrode locations for the TA, GcM, and Sol were the midportion of the muscle belly. After shaving and cleaning the skin with alcohol, electrodes were aligned along the major axis of each muscle with an inter-electrode distance of approximately 3 cm. All electrode input impedance was reduced to ≤5 kΩ. Signals from electrodes were amplified (EEG, ×40,000; EOG, ×4000; and EMG, ×4000) and band-pass filtered (EEG, 0.05–100 Hz; EOG, 0.05–30 Hz; and EMG, 5–500 Hz) using an amplifier (6R12, NEC-Sanei, Japan).

The position of the body in the sagittal plane was recorded using the Position Sensor System (C5949, Hamamatsu Photonics, Japan). This system is composed of light-emitting diode (LED) targets and a sensor head and emits analog outputs of the coordinates of the LED targets in two dimensions. The LED targets were placed over the vertebra prominens (C7), greater trochanter, knee (lateral condyle of femur), and lateral malleolus on the right side of the body. The sensor head was placed 4 m away from the right side of the subject.

All recorded signals were sent to a computer (Dimension 9150, DELL, Japan) via an A/D converter (AD16-64(LPCI)LA, Contec, Japan) with a 1000-Hz sampling rate and 16-bit resolution. The signal from the force platform was sent to another online computer (PC9801CV21, NEC, Japan) via an A/D converter (PIO9045, I/O Data, Japan) with a 20-Hz sampling rate and 12-bit resolution.

### Fixation of the leg and trunk

Before the experiment, a cast brace that immobilized the joints in the leg and trunk except the ankle was made for each subject. The brace was fitted while the subject was maintaining a quiet standing posture. First, while the subject was wearing a t-shirt and tights, hydraulic casting tape (CASTLIGHT-α, ALCARE, Japan) was wrapped around the body from the level of first thoracic vertebra to just above the lateral malleolus. After the cast hardened, it was divided into front and rear parts using a cutter. The cast brace had seven openings (5 cm × 8 cm) for the EMG electrode cables and was made within an hour. During the experiment, the cast was secured to the subject using a bandage (ELASCOT, ALCARE, Japan) (Fig. 1b).

### Procedure

Subjects stood on the platform with bare feet 10 cm apart and parallel. They were instructed to maintain a standing posture with their arms relaxed by their sides and eyes closed and not to intentionally flex their knees or trunk during the oscillation. To avoid fluctuations in EEG due to eye movements, subjects were also instructed to minimize eye movement even when they had their eyes closed. The floor oscillation task was performed in two conditions: (1) without joint fixation (no-fixation condition) and (2) with joint fixation by the cast brace (fixation condition). The no-fixation condition was always performed first. Subjects maintained a quiet standing posture for 10 s on a stationary floor, and then 60 s of floor oscillation occurred, which was defined as one trial. The initial movement of the floor was always in the forward direction. Trials were repeated until the subject sufficiently adapted to the floor oscillation, defined using criteria described in a previous study [11]. At least five trials were performed in each condition. To eliminate the effects of fatigue on postural muscle and brain activity, subjects rested for 60 s between trials. During the rest, subjects sat on a chair in the no-fixation condition and were supported by an experimenter or alternately maintained forward and backward leaning postures several times in the fixation condition. After all trials were completed, maximum voluntary isometric contractions (MVC) of each muscle under study were performed for 3 s while sitting on the chair.

### Data analyses

**Fig. 2:**
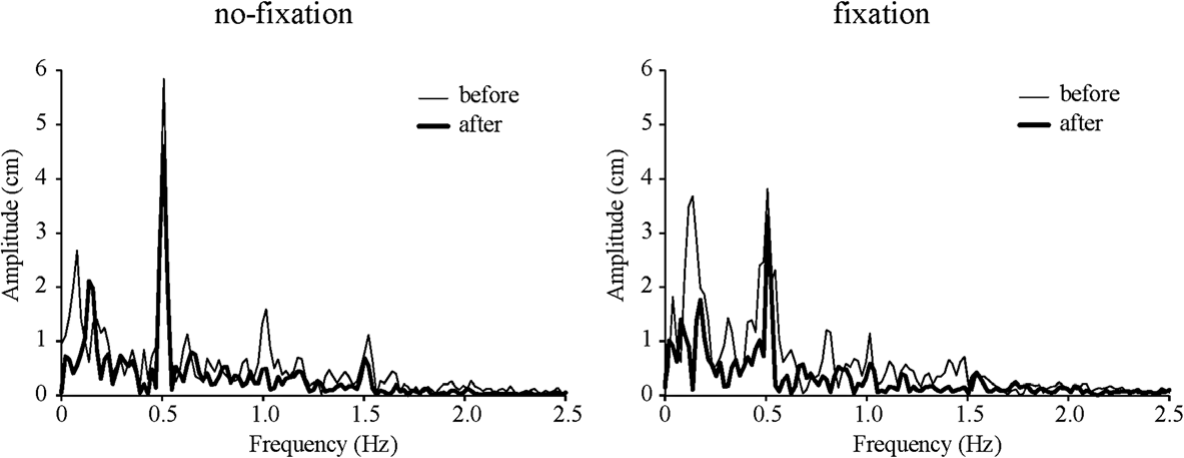
Typical frequency spectrum of CoPap fluctuation during floor oscillations in no-fixation (*left*) and fixation (*right*) conditions. *Thin* and *thick lines* indicate before and after adaptation, respectively

In each trial, data from 10 to 60 s after the onset of the table oscillation were analyzed. The initial 10 s of data were excluded to eliminate any transient changes in acceleration induced by the onset of table oscillation [11]. The mean speed of CoPap fluctuation (CoPap speed) during the floor oscillation was calculated online using CoPap data smoothed by a weighted five-point moving average [6]. This moving average corresponded to a 1.8-Hz low-pass filter. We confirmed that the main frequencies of the body sway with a 20-Hz sampling rate during the floor oscillation were less than 1.8 Hz (Fig. 2).

The following analyses for EEG, EOG, EMG, CoPap, and position sensor data were performed using BIMUTAS II software (Kissei Comtec, Japan). In the no-fixation and fixation conditions, data from the first and second trials were analyzed as “before adaptation” and data from the last two trials were analyzed as “after adaptation.” The EEG, EOG, EMG, and CoPap waveforms were extracted from 1500 ms before each anterior reversal to 500 ms after the reversal (i.e., one cycle of floor oscillation) without overlapping with the next period. The extracted period was defined as a data segment. For EEG and EOG, the mean amplitude of the data in each 2000-ms segment was defined as baseline, and segments in which the amplitude of EOG and/or EEG exceeded ±100 μV were discarded. EMG data were high-pass filtered at 40 Hz using a seventh-order Butterworth filter to exclude electrocardiographic and movement artifacts and then full-wave rectified. EEG, EOG, EMG, and CoPap waveforms from 20–50 cycles of floor oscillation were obtained and averaged in each adaptation state (before adaptation, after adaptation) and condition (no-fixation, fixation) for each subject. These averaged waveforms were used for the following analyses.

For CoPap, the mean amplitude of the averaged waveform was calculated and expressed as a percentage distance from the heel in relation to the FL (%FL). The averaged EEG, EOG, and rectified EMG (rEMG) waveforms were smoothed by a 111-point moving average, corresponding to a 4-Hz low-pass filter. For EEG, the positive and negative peaks were identified, and peak-to-peak amplitude was calculated as ERP amplitude. For all subjects, the maximum ERP amplitude before adaptation in the no-fixation condition was obtained at Cz. Thus, EEG waveforms recorded from Cz were analyzed. For EOG, peak-to-peak amplitude and cross-correlation coefficients with ERP waveform were calculated. For rEMG, the maximum increase from baseline was defined as rEMG peak amplitude and was normalized to the mean rEMG value over 1 s of the MVC (%MVC). The time of the negative peak of the ERP and the peak of the rEMG was calculated relative to the time of the anterior reversal and is referred to as peak time. The peak time is represented as positive if it was after the anterior reversal and negative if it was before the anterior reversal point.

Spatial coordinate signals from each body part were smoothed by an 89-point moving average, corresponding to a 5-Hz low-pass filter. The *x*- and *y*-coordinates of the LED targets were used to calculate hip angle (knee − greater trochanter − C7), knee angle (lateral malleolus − knee − greater trochanter), and ankle angle (inclination of lateral malleolus − knee from vertical line) in Excel 2010 software (Microsoft, Japan). Joint angles were calculated for every data point, and the waveform was then averaged in the same way as described for CoPap. The movement angle of each joint was defined as the difference between maximal and minimal values of the averaged waveform.

### Statistical analysis

Shapiro-Wilk tests confirmed that all data satisfied the assumptions of normality. One-way repeated-measures analysis of variance (ANOVA) was used to compare CoPap speed across the first five trials in each condition (no-fixation, fixation). Post hoc comparisons were performed using Tukey’s honestly significant difference (HSD) test to further examine differences suggested by ANOVA. Two-way repeated-measures ANOVA was used to assess the effects of condition (no-fixation, fixation) and adaptation state (before adaptation, after adaptation) on CoPap speed, mean CoPap position, and peak amplitudes and peak times of rEMG and ERP. When a significant interaction was present, paired *t* tests were used to investigate differences within each factor. Three-way ANOVA was used to assess the effects of condition (no-fixation, fixation), adaptation state (before adaptation, after adaptation), and joint (hip, knee, ankle) on joint movement angle. When a significant interaction was present, post hoc Tukey’s HSD test or a paired *t* test was used to assess the differences within each factor. A paired *t* test was used to compare each parameter across the after-adaptation trials in the no-fixation condition and the before-adaptation trials in the fixation condition. One sample *t* test was used to assess whether the peak times of ERP and rEMG significantly differed from the anterior reversal point of the floor oscillation. Pearson correlations were used to assess the relations between the peak times of the negative ERP and rEMG of each muscle. The alpha level was set at *p* < 0.05. All statistical analyses were performed using IBM SPSS Statistics 19 (IBM Japan, Japan).

## Results

The statistical results of the two- and three-way ANOVAs are shown in Tables 1 and 2, respectively. The results of the post hoc tests are described in the text with the corresponding *p* values.

**Table 1:** Results of two-way ANOVA

Dependent variables	Condition		Adaptation		Interaction	
	*F* value	Significance	*F* value	Significance	*F* value	Significance
CoPap speed	*F*_1,11_ = 4.6	n.s.	*F*_1,11_ = 48.3	*p* < 0.001	*F*_1,11_ = 5.6	*p* < 0.05
CoPap position	*F*_1,11_ = 2.3	n.s.	*F*_1,11_ = 7.3	*p* < 0.05	*F*_1,11_ = 2.5	n.s.
Peak time of ES	*F*_1,11_ = 10.6	*p* < 0.01	*F*_1,11_ = 3.4	n.s.	*F*_1,11_ = 0.1	n.s.
Peak time of BF	*F*_1,11_ = 0.3	n.s.	*F*_1,11_ = 0.5	n.s.	*F*_1,11_ = 0.1	n.s.
Peak time of GcM	*F*_1,11_ = 0.2	n.s.	*F*_1,11_ = 0.6	n.s.	*F*_1,11_ = 0.8	n.s.
Peak time of Sol	*F*_1,11_ = 0.1	n.s.	*F*_1,11_ = 0.2	n.s.	*F*_1,11_ = 1.1	n.s.
Peak time of negative ERP	*F*_1,11_ = 0.1	n.s.	*F*_1,11_ = 0.3	n.s.	*F*_1,11_ = 0.2	n.s.
Peak amplitude of RA	*F*_1,11_ = 0.3	n.s.	*F*_1,11_ = 14.8	*p* < 0.01	*F*_1,11_ = 1.0	n.s.
Peak amplitude of ES	*F*_1,11_ = 4.5	n.s.	*F*_1,11_ = 10.4	*p* < 0.01	*F*_1,11_ = 2.3	n.s.
Peak amplitude of Rf	*F*_1,11_ = 3.1	n.s.	*F*_1,11_ = 2.7	n.s.	*F*_1,11_ = 1.8	n.s.
Peak amplitude of Bf	*F*_1,11_ = 0.2	n.s.	*F*_1,11_ = 4.9	*p* < 0.05	*F*_1,11_ = 6.9	*p* < 0.05
Peak amplitude of TA	*F*_1,11_ = 11.8	*p* < 0.01	*F*_1,11_ = 17.9	*p* < 0.01	*F*_1,11_ = 4.3	n.s.
Peak amplitude of GcM	*F*_1,11_ = 0.4	n.s.	*F*_1,11_ = 20.5	*p* < 0.01	*F*_1,11_ = 1.4	n.s.
Peak amplitude of Sol	*F*_1,11_ = 4.1	n.s.	*F*_1,11_ = 13.2	*p* < 0.01	*F*_1,11_ = 2.1	n.s.
ERP amplitude	*F*_1,11_ = 0.3	n.s.	*F*_1,11_ = 6.4	*p* < 0.05	*F*_1,11_ = 1.2	n.s.

*ANOVA* analysis of variance, *n.s.* not significant

**Table 2:** Results of Three-way ANOVA for joint movement angle

	*F* value	Significance
Condition	*F*_1,33_ = 42.4	*p* < 0.001
Adaptation	*F*_1,33_ = 12.8	*p* < 0.01
Joint	*F*_2,33_ = 25.7	*p* < 0.001
Condition × adaptation	*F*_1,33_ = 7.1	*p* < 0.05
Condition × joint	*F*_2,33_ = 29.4	*p* < 0.001
Adaptation × joint	*F*_2,33_ = 2.0	n.s.
Condition × adaptation × joint	*F*_2,33_ = 2.1	n.s.

*ANOVA* analysis of variance, *n.s.* not significant

**Fig. 3:**
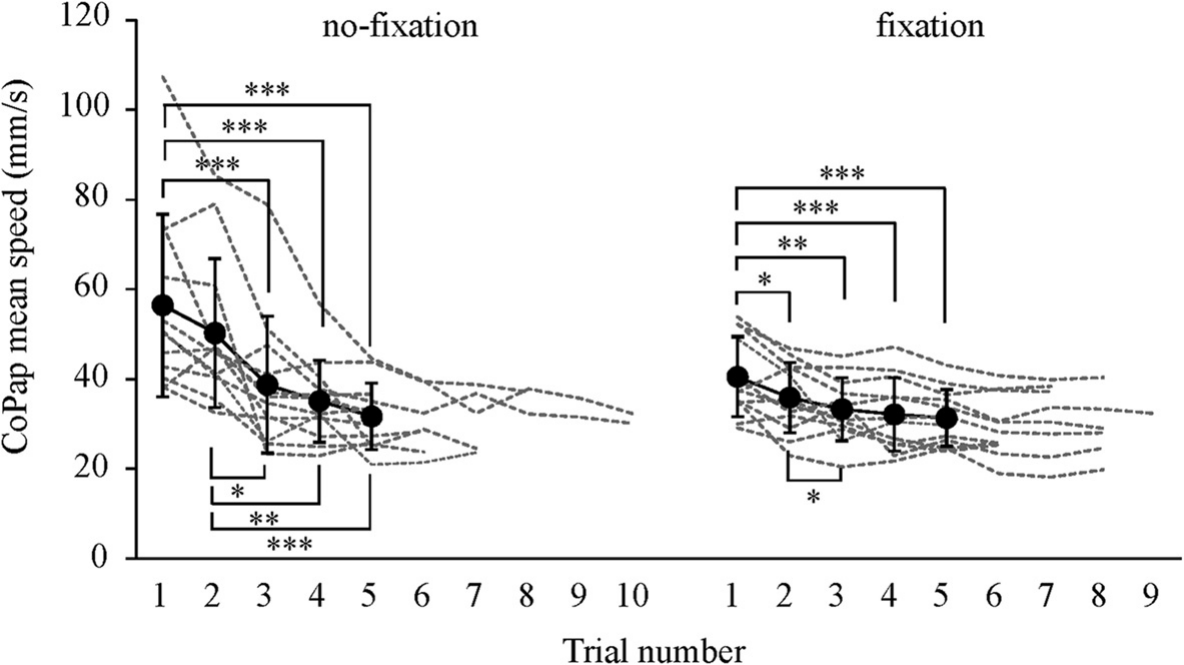
Mean speed of CoPap fluctuation during floor oscillations. *Filled circles* and *solid lines* indicate the average and standard deviation, respectively, in each trial until the fifth trial. *Dashed lines* indicate the mean speed of CoPap fluctuation over all trials for each subject. *p < 0.05; **p < 0.01; ***p < 0.001

CoPap speed significantly decreased with trial repetition in both fixation conditions (no-fixation: *F*_4,44_ = 16.0, fixation: *F*_4,44_ = 12.5, both *p* < 0.001; Fig. 3). The number of trials required for adaptation was 6.8 (2.0) trials (range, 5–10 trials) in the no-fixation condition and 7.2 (1.2) trials (range, 5–9 trials) in the fixation condition, with no significant difference between the two conditions. The CoPap speed before adaptation in the fixation condition (38.5 (8.0) mm/s) was significantly larger than that after adaptation in the no-fixation condition (30.2 (5.1) mm/s, *p* < 0.01). The mean CoPap position shifted backward after adaptation regardless of fixation condition (no-fixation: before adaptation, 51.2 (7.6) %FL; after adaptation, 47.8 (7.0) %FL; fixation: before adaptation, 48.0 (2.8) %FL; after adaptation 46.8 (3.3) %FL, *p* < 0.05).

**Fig. 4:**
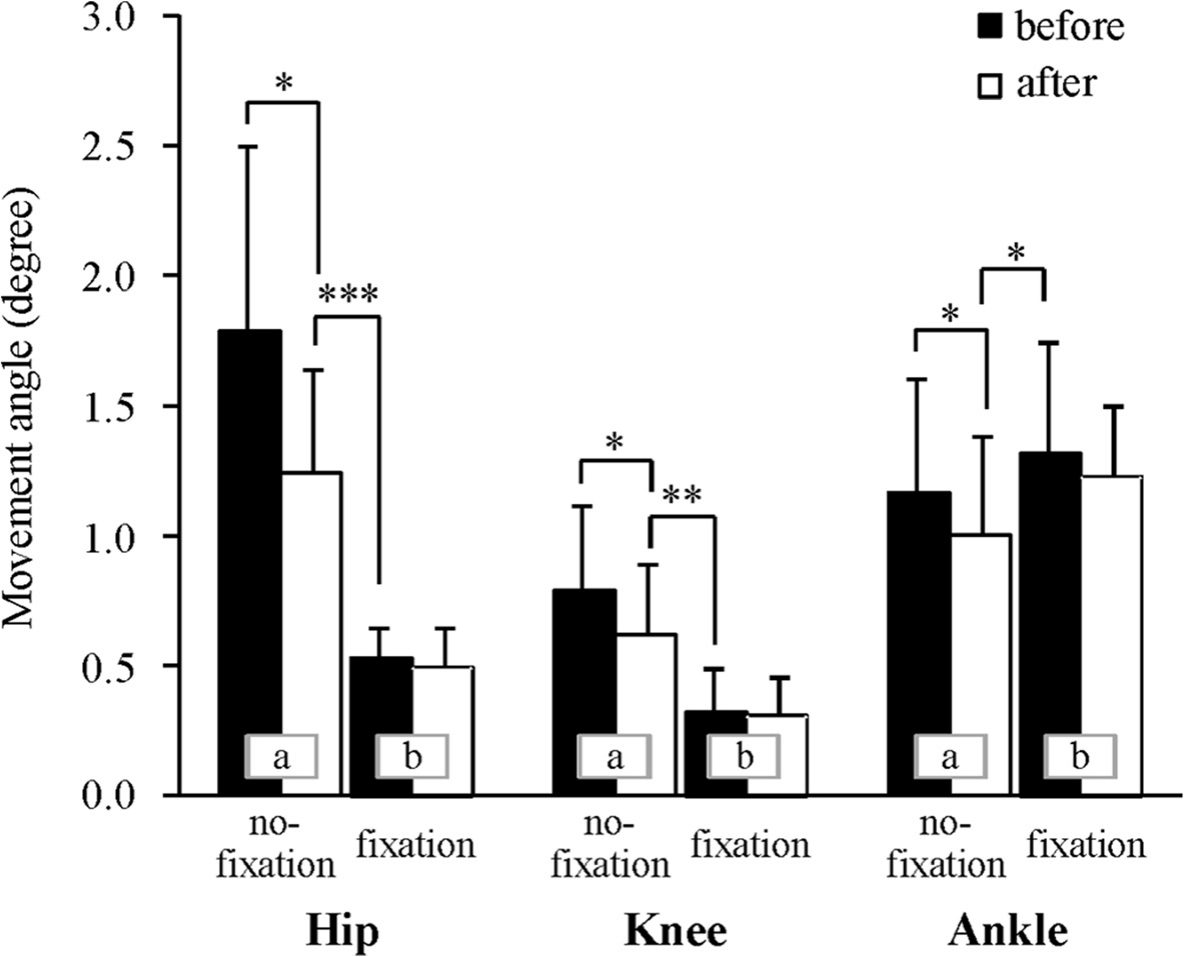
Mean and standard deviation joint movement angle. *Letters* in the bar graph indicate a significant difference between same letter. *p < 0.05; **p < 0.01; ***p < 0.001

Joint movement angles are shown in Fig. 4. In the no-fixation condition, the joint movement angle was largest in the hip, followed by the ankle and then the knee, and was lower after adaptation than before adaptation in all three joints (*p* < 0.05). The movement angle of the hip and knee was much lower in the fixation condition than in the no-fixation condition, regardless of adaptation (about 45 and 60 % of the angle after adaptation in the no-fixation condition, respectively, *t*_11_ > 3.6, both *p* < 0.01), whereas that of the ankle was greater (about 140 %, *t*_11_ = 2.6, *p* < 0.05). The movement angle after adaptation was similar to that before adaptation for all joints.

**Fig. 5:**
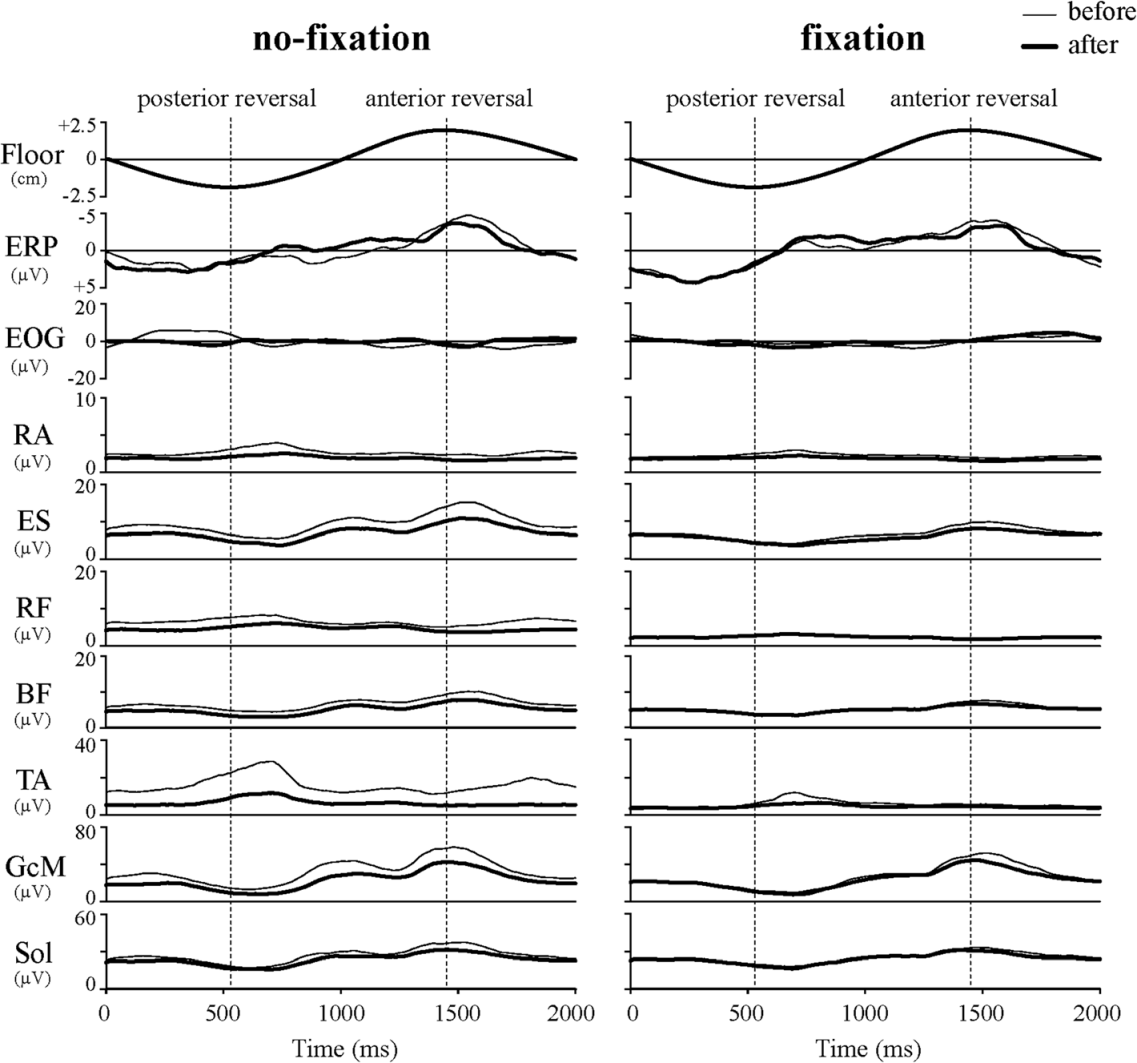
Grand-averaged ERP and rEMG waveforms in the no-fixation and fixation conditions. *Thin lines* and *bold lines* indicate trials before and after adaptation, respectively

**Fig. 6:**
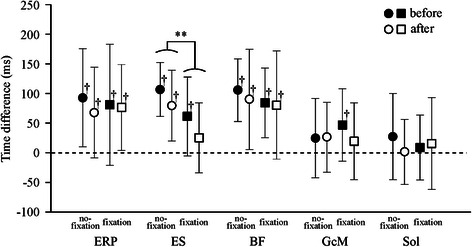
Peak time of the ERP and rEMG with respect to the anterior reversal of floor oscillation. **p < 0.01; ^†^significant difference from time of anterior reversal

Grand-averaged rEMG waveforms are shown in Fig. 5. The maximum activity of the anterior postural muscles occurred near the posterior reversal point. For the posterior postural muscles, the maximum activity occurred near the anterior reversal point and the minimum occurred near the posterior reversal point. The peak time of GcM and Sol was not significantly different from the anterior reversal point, except for GcM before adaptation in the fixation condition (Fig. 6). The peak time of ES was significantly earlier in the fixation condition than in the no-fixation condition, regardless of adaptation. After adaptation in the fixation condition, the peak time of ES was not significantly different from the anterior reversal point, resulting in simultaneous peak times of ES, GcM, and Sol.

**Fig. 7:**
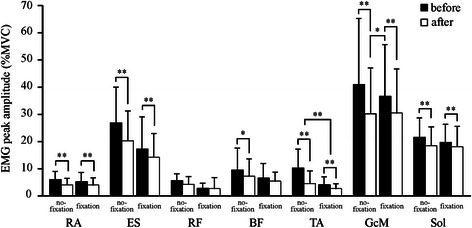
Mean and standard deviation rEMG peak amplitude. *p < 0.05; **p < 0.01

Peak rEMG amplitude was largest in GcM (30–40 % MVC), followed by ES and Sol (Fig. 7), regardless of adaptation and fixation condition. The peak amplitude of the anterior muscles was less than 10 % MVC. In the no-fixation condition, the peak amplitude for all muscles except RF significantly decreased with adaptation (before vs. after adaptation, all *p* < 0.05). The peak amplitude of GcM before adaptation in the fixation condition was significantly greater than that after adaptation in the no-fixation condition (*t*_11_ = 2.4, *p* < 0.05). The peak amplitude of RA, ES, TA, GcM, and Sol significantly decreased with adaptation (before vs. after adaptation, all *p* < 0.01).

Grand-averaged ERP and EOG waveforms are shown in Fig. 5. In the EOG waveforms, the peak-to-peak amplitudes were extremely small (mean 27.9 [20.4] μV). Cross-correlation coefficients between ERP and EOG were very low (mean *Z* value 0.15 [0.56]). These findings indicate no influence of EOG on ERP. The ERP had a positive peak about 100 ms before the posterior reversal of the oscillation and gradually became more negative toward the anterior reversal. This negative shift became sharp about 100 ms before the anterior reversal and peaked about 80 ms after the anterior reversal. The peak time of the negative ERP was similar across conditions and adaptation states (Fig. 6). ERP amplitude significantly decreased with adaptation, regardless of fixation condition (no-fixation: before adaptation, 10.8 [4.6] μV; after adaptation, 8.3 [2.9] μV; fixation: before adaptation, 10.0 [3.9] μV; after adaptation, 9.3 [2.7] μV; *p* < 0.05).

**Fig. 8:**
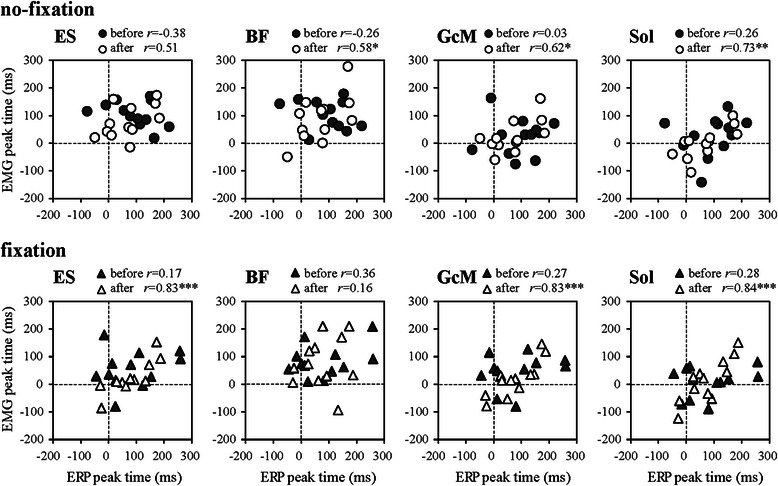
Relations between peak times of ERP and rEMG

After adaptation in the no-fixation condition, the peak time of the negative ERP was significantly correlated with the peak time of BF (*r* = 0.58), GcM (*r* = 0.62), and Sol (*r* = 0.73) (all *p* < 0.05; Fig. 8). After adaptation in the fixation condition, the peak time of the negative ERP was significantly and strongly correlated with the peak time of ES (*r* = 0.83), GcM (*r* = 0.83), and Sol (*r* = 0.84) (all *p* < 0.01). No significant correlation was found before the adaptation in either condition.

## Discussion

In the no-fixation condition, CoPap speed adaptively decreased until the third trial and reached a plateau between the fifth and tenth trials. After this adaptation, all joints of legs and trunk except the ankles were fixed to investigate postural control when pivoting at the ankle. CoPap speed increased just after joint fixation, but the improvement in postural control with repeated trials was similar to that in the no-fixation condition.

The adaptive changes in joint movement range and postural muscle activity in the no-fixation condition were consistent with previous reports [8, 11]. The joint movement range was larger at the ankle and hip than at the knee, and postural muscle activities were the largest in the TS followed by the ES. With adaptation, the movement range of all joints and activities of all posterior postural muscles markedly decreased. In the fixation condition, hip and knee movements were considerably limited, while movement at the ankle significantly increased. Only GcM activity significantly increased just after the fixation. These results indicate that the knees, hips, and trunk, particularly the knees, were rigidly fixed and that the ankles were free and that postural control pivoting at the ankle was mainly focused on the TS, especially the GcM. The activity of ES did not significantly decrease with the fixation. As the trunk contains the viscera, we ensured that the fixation brace did not exceed abdominal pressure or limit breathing. As such, it was difficult to fix the trunk tightly, and some mobility of the spinal column likely remained in the fixation condition, leading to some activity of the ES [20]. With adaptation in the fixation condition, the activity of the muscles around the ankle joint, especially the GcM, significantly decreased, but joint movement ranges did not change. The decrease in GcM activity is likely related to the backward shift in the mean CoPap position that occurred with adaptation in this condition. Furthermore, ES peak simultaneously occurred with GcM and Sol peak and anterior reversal of floor oscillation. The creation of a rigid body by the fixation would result in a small phase difference between body segments.

ERP waveforms showed the same pattern as in our previous study [11], regardless of the fixation condition. In the averaged waveform of EOG, the changes in EOG amplitude were extremely small, indicating that few movements of the eyes were involved. Head rotation in the vertical direction was followed by eye movements in the same direction [12]. Therefore, this EOG averaged waveform also demonstrates that there was little head rotation, suggesting a small effect on the vestibular organ. The negative peak of the ERP that appeared 100 ms after the postural perturbation, termed N1, is considered to reflect the processing of sensory information related to the postural disturbance [14–16]. In addition, changes in the negative peak of ERP during floor oscillation (with an 80-ms delay to the anterior reversal) would reflect changes in attention [11]. In this study, positive correlations between peak times of the negative ERP and muscle activity were observed only after adaptation, regardless of the fixation condition. In the no-fixation condition, the peak time of the negative ERP was correlated with the peak time of all posterior muscles except the ES (*r* = 0.58–0.73), and only the TS peak preceded the ERP peak (by about 70 ms). In the fixation condition, the correlation between the peak time of the negative ERP and the TS was stronger (*r* = 0.83–0.84) than in the no-fixation condition. Furthermore, there was a correlation between the peak time of the negative ERP and the ES (*r* = 0.84), which did not exist in the no-fixation condition. These activities of the GcM, Sol, and ES peaked at the same time as the anterior reversal and about 60 ms earlier than the negative ERP peak. Phase differences between the activity timings of the main postural muscles without fixation would lead to the phase differences of muscle sensory information. Therefore, sensory information to which attention was directed varied by individual, which would result in a relatively low correlation between the peak times of negative ERP and TS. This tendency remained just after the fixation. However, after adaptation in the fixation condition, because attention could be focused on the sensory information processing related to the TS in many subjects, an extremely high correlation was obtained between the peak times of negative ERP and TS. As mentioned above, ES also showed high correlation with ERP peak time in the fixation condition, suggesting that the attention may be directed also to the sensory information related to the ES. However, the small ES activity and hip mobility in the fixation condition suggest that it is unlikely possible that the attention was strongly directed to the ES.

ERP amplitude decreased with adaptation, regardless of the fixation condition. This result is consistent with our previous report [11]. Quant et al. [21] observed that the magnitude of N1 response to a mechanical perturbation significantly decreased in a case where the perturbation was accompanied with cognitive visuo-motor tracking task and considered that the N1 amplitude would reflect the amount of attention to the processing of sensory information related to postural perturbation. Adkin et al. [22] reported that N1 amplitude following postural perturbation significantly increased under high anxiety conditions such as standing at high altitude, suggesting that attention was more likely to be directed to the perturbation. Mochizuki et al. [16] reported that N1 response attenuated when the timing of the external perturbation was predictable. Therefore, the adaptive decrease in ERP amplitude in the present study would suggest decreases in the necessity of postural preparation for the anterior reversal and in the attention to somatosensory information near the reversal [11].

In future studies, to confirm the abovementioned hypotheses, by applying the electrical and mechanical stimuli to the muscle, vestibular organ, and skin during floor oscillation, we will investigate the detailed relation between ERP waveform and attention to the sensory information.

## Conclusions

Joint fixation of the knee, hip, and trunk increased the rigidity of the whole body except the ankle and resulted in simultaneous activation of the TS and ES during standing on an oscillating table. After adaptation of postural control pivoting at the ankle, the peak time of TS and ES muscle activation was more strongly correlated with the peak time of the negative ERP than it was before the fixation. Attention would be more focused on processing the muscle sensory information from the TS and/or ES, especially that from GcM, which had the largest activation.

## Abbreviations

ANOVA: analysis of variance
BF: biceps femoris
C7: vertebra prominens
CoPap: center of foot pressure in the anteroposterior direction
CoPap speed: mean speed of CoPap fluctuation
EEG: electroencephalogram
EMG: electromyogram
EOG: electrooculogram
ERP: event-related brain potential
ES: erector spinae
FL: foot length
GcM: gastrocnemius
HSD: honestly significant difference
LED: light-emitting diode
MVC: maximum voluntary isometric contraction
RA: rectus abdominis
rEMG: rectified electromyogram
RF: rectus femoris
Sol: soleus
TA: tibialis anterior
TS: triceps surae
